# Trends in US Emergency Department Use After Sexual Assault, 2006-2019

**DOI:** 10.1001/jamanetworkopen.2022.36273

**Published:** 2022-10-20

**Authors:** Emily L. Vogt, Charley Jiang, Quinton Jenkins, Maya J. Millette, Martina T. Caldwell, Kathleen S. Mehari, Erica E. Marsh

**Affiliations:** 1 University of Michigan Medical School, University of Michigan, Ann Arbor; 2 Division of Reproductive Endocrinology and Infertility, Department of Obstetrics and Gynecology, University of Michigan, Ann Arbor; 3 School of Public Health, University of Michigan, Ann Arbor; 4 Department of Emergency Medicine, Henry Ford Hospital, Detroit, Michigan; 5 Division of Women’s Health, Department of Obstetrics and Gynecology, University of Michigan, Ann Arbor

## Abstract

**Importance:**

Adult sexual assault (SA) survivors experience numerous emergent health problems, yet few seek emergency medical care. Quantifying the number and types of survivors presenting to US emergency departments (EDs) after SA can inform health care delivery strategies to reduce survivor morbidity and mortality.

**Objective:**

To quantify ED use and factors that influenced seeking ED care for adult SA from 2006 through 2019.

**Design, Setting, and Participants:**

This cross-sectional study used SA data from the Nationwide Emergency Department Sample from 2006 through 2019, which includes more than 35.8 million observations of US ED visits from 989 hospitals, a 20% stratified sample of hospital-based EDs. The study also used the Federal Bureau of Investigation’s Uniform Crime Reporting Program, which includes annual crime data from more than 18 000 law enforcement agencies representing more than 300 million US inhabitants. The study sample included any adult aged 18 to 65 years with an ED visit in the Nationwide Emergency Department Sample coded as SA. The data were analyzed between January 2020 and June 2022.

**Main Outcomes and Measures:**

Annual SA-related ED visits, subsequent hospital admissions, and associated patient-related factors (age, sex, race and ethnicity, income quartile, and insurance) were analyzed using descriptive statistics.

**Results:**

Data were from 120 to 143 million weighted ED visits reported annually from 2006 through 2019. Sexual assault–related ED visits increased more than 1533.0% from 3607 in 2006 to 55 296 in 2019. Concurrently, admission rates for these visits declined from 12.6% to 4.3%. Female, younger, and lower-income individuals were more likely to present to the ED after SA. Older and Medicaid-insured patients were more likely to be admitted. Overall, the rate of ED visits for SA outpaced law enforcement reporting.

**Conclusions and Relevance:**

This cross-sectional study found that US adult SA ED visits increased from 2006 through 2019 and highlighted the populations who access emergency care most frequently and who more likely need inpatient care. These data can inform policies and the programming needed to support this vulnerable population.

## Key Points

**Question:**

What are the trends and disparities in US emergency department use by adult sexual assault survivors from 2006 to 2019?

**Findings:**

This cross-sectional study of 120 to 143 million weighted emergency department visits made annually from 2006 through 2019 revealed a significant 1533.0% increase in sexual assault emergency department visits, outpacing the growth of law enforcement reporting. Concurrently, there was a significant 8.3% decrease in admission rates for sexual assault.

**Meaning:**

Seeking emergency medical help after sexual assault increased markedly in the past decade and warrants expansion of outpatient health care support for adult sexual assault survivors.

## Teaser

This cross-sectional study quantifies and describes national US emergency department use by adult sexual assault survivors.

## Introduction

Sexual assault (SA) is a disturbing and prevalent issue in the US, with a new assault occurring every 68 seconds.^1^ Based on data from the Federal Bureau of Investigation (FBI), national reported rapes and SAs have increased from 93 000 in 2006 to 139 815 in 2019, peaking at 143 765 in 2018.^2^ Studies have revealed that SA survivors are at greater risk for suicidal ideation, posttraumatic stress disorder, depression, substance use, and chronic conditions than their peers who have not experienced SA.^3,4,5,6,7,8^ These sequelae are complicated by previous findings that SA survivors are less likely to seek formal health services than survivors of other crimes,^9,10,11^ although this association has not been quantified on a national scale.

Emergency departments (EDs) have long been viewed as the primary point of care for SA survivors seeking medical services because of the acute nature of these traumas and the stigma surrounding other care avenues.^12,13,14^ Currently, more than 800 US SA forensic nurse examiner programs have been established, many of which are ED affiliated.^15^ These programs improve SA care with better documentation, evidence collection, treatment protocols, and emotional support than other sites.^16,17,18,19^ For this reason, EDs have become the preferred setting for evaluating current trends in SA care delivery.^20,21,22^

Previous studies have shown that survivors presenting to the ED most often are younger, are White, have a known assailant, and have physical evidence of trauma.^13,21,23,24^ The longest national study to date showed that SA comprised 4.4% of ED visits for violence and had an average hospital admission rate of 2.7%.^21^ A minority of survivors seek medical care, with 1 study estimating that only 21% of survivors will seek any type of care.^10^ Unfortunately, survivors often receive inadequate or incomplete care.^23,25^ Overall, studies in the existing literature have been limited to a single population,^23,26,27,28^ location,^13,29,30,31^ or year.^25,32^ Current gaps in the literature include lack of commentary on socioeconomic and insurance-related variables and lack of longitudinal studies. The primary objective of this study was to quantify and describe national US ED use by adult SA survivors from 2006 through 2019, with a goal of expanding on the existing literature and identifying areas of health disparity and opportunities for reform.

## Methods

### Study Design

This cross-sectional study leveraged data from 2 national databases. Using data from the Nationwide Emergency Department Sample (NEDS), we analyzed ED use for adult SA-related diagnoses and hospital charges from 2006 through 2019. We then compared trends in SA ED use with national trends in FBI-reported SA using data from the Uniform Crime Reporting Program (UCRP). The period from 2006 through 2019 was selected because these are the years for which NEDS currently has available data. Because the data used in this study are deidentified and publicly available, the University of Michigan institutional review board deemed it exempt from review and the requirement for informed consent. This study followed the Strengthening the Reporting of Observational Studies in Epidemiology (STROBE) reporting guideline.^33^

### Data Sources and Setting

The NEDS is the largest nationally representative ED database and is managed by the Healthcare Cost and Utilization Project (HCUP) of the Agency for Healthcare Research and Quality. It includes more than 35.8 million observations of US ED visits from 989 hospitals across 40 states, representing a 20% stratified sample of hospital-owned EDs.^34^ State participation varies on the basis of voluntary contributions. The NEDS contains information found in a typical discharge summary, with 100 clinical and nonclinical variables for each hospital stay, including *International Classification of Diseases, Ninth Revision* (*ICD-9*) and *Tenth Revision* (*ICD-10*) codes; discharge status; patient demographic characteristics; hospital characteristics; expected payment source; total ED charges; and total hospital charges.

The UCRP is a publicly available, FBI-operated database. It includes reported offense and violence exposure data voluntarily contributed by 18 000 law enforcement agencies (LEAs), representing more than 300 million US inhabitants in 2010.^35^ Reported data include state-by-state arrests, clearances, trends, and law enforcement employee data.^35^ The UCRP counts offenses only, making it possible to doubly count repeat offenders and individuals who reported SA.

### Participants

Participants included adults aged 18 to 65 years seen in the ED with any *ICD-9* or *ICD-10* code of SA from January 1, 2006, through December 31, 2019. If *ICD-9* code 995.83^36^ or *ICD-10* codes T74.21XA,^37^ T76.21,^38^ T76.21XA,^39^ or T74.51XA^40^ were in any diagnostic field, that visit was included. *ICD-9* codes were reported in NEDS from 2006 through the third quarter of 2015, whereas *ICD-10* codes were reported from the fourth quarter of 2015 through 2019.^41,42^ Sample sizes smaller than 11 were excluded from the analysis per HCUP requirements. Visits with missing data or without an *ICD*-coded diagnosis of SA were excluded. The final sample of 120 to 130 million weighted visits annually was based on eligibility criteria.

### Measures

Sexual assault was defined using *ICD* codes from NEDS data. There is 1 *ICD-9* code for adult SA (995.83), whereas *ICD-10* coding allows for suspected SA (T76.21XA), confirmed SA (T74.21XA), or forced penetration (T74.51XA). The FBI’s definitions of rape and SA were used for UCRP data. In 2013, the FBI adapted its definition for SA from “the carnal knowledge of a female forcibly and against her will” to “penetration, no matter how slight, of the vagina or anus with any body part or object, or oral penetration by a sex organ of another person, without the consent of the victim.”^43^ The former is now known as the legacy definition and the latter as the revised definition. We included data from both FBI definitions in our analysis to account for this discrepancy.

Primary analysis measures for NEDS included ED visits and hospital admissions. Secondary analysis measures included age, sex, income quartile by zip code, and payment method. Race and ethnicity were analyzed for 2019 (the first year NEDS contained these data), given their relevance for highlighting potential racial disparities as they relate to ED use for SA. Per HCUP data collection, race and ethnicity categories were Asian or Pacific Islander, Black, Hispanic, Native American, White, or other.^44^ Other was defined as any individual identifying with multiple categories or with another group not delineated. Total annual and average annual inflation-adjusted hospital charges were also analyzed.

### Statistical Analysis

Statistical analyses were conducted between January 2020 and June 2022. Descriptive statistics were calculated as counts and percentages for categorical variables and as means and SDs for continuous variables. χ^2^, *t*, and *F* tests were performed to assess for statistical significance. All-cause ED visits were added as a comparator to SA ED visits. Inflation-adjusted analysis of average annual and total annual hospital charge data for SA ED visits was performed relative to the 2019 US dollar. A joinpoint regression model was used to perform the trend analysis of hospital charge data. Average annual percentage changes (AAPCs) of charges were estimated by fitting trend data to a log-linear model. Missing data were handled by imputation for calculation of total charges, with age, region, income, and any SA-related diagnosis as covariates. Analyses were conducted using SAS, version 9.4 (SAS Institute) and Joinpoint, version 4.7.0.0 (National Cancer Institute) statistical software, and a 2-sided *P* < .05 was considered statistically significant.

## Results

**Figure 1.**
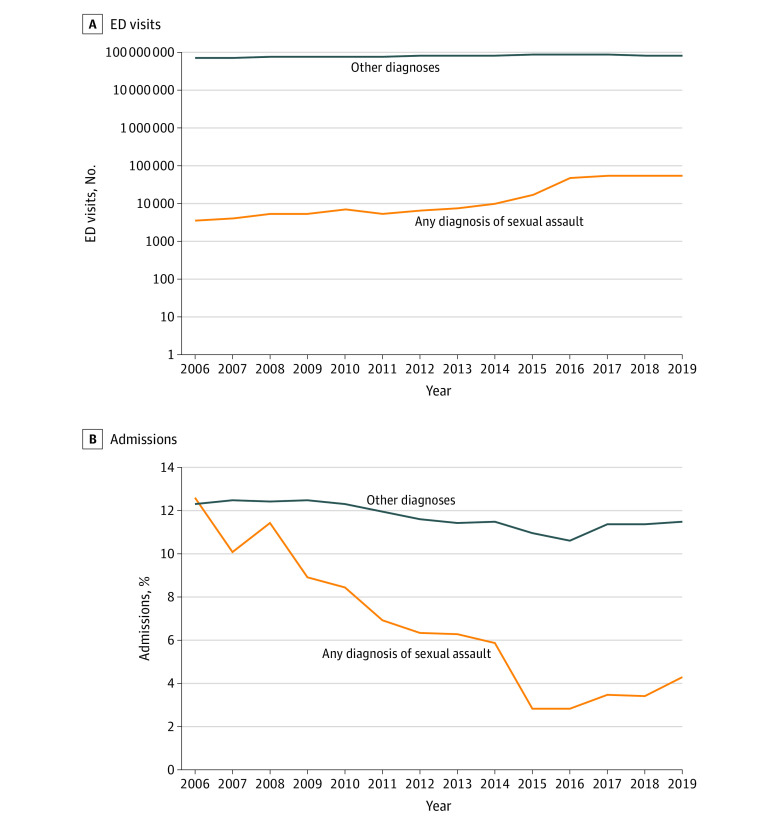
Emergency Department (ED) Visits and Admissions for Adults Aged 18 to 65 Years With Any Diagnosis of Sexual Assault vs All Other Diagnoses, 2006-2019

Our analysis revealed that US ED visits for SA increased by more than 1533.0% from 3607 visits in 2006 to 55 296 visits in 2019 (vs a 21.4% increase in all-cause ED visits) (Figure 1), with an AAPC rate of 23.0% (range, 14.5%-32.0%; *P* < .001). This increase was most notable from 2015 to 2016, when annual visits increased from 17 709 to 47 732. Analysis of *ICD* coding from October 2015 (after establishment of *ICD-10*) through December 2019 revealed that 50.7% (25 149-28 865 visits annually) of SA ED visits were coded as suspected adult rape/SA, 49.3% (22 584-29 542 visits annually) as confirmed adult rape/SA, and 0.05% (0-108 visits annually) as adult forced sexual exploitation. In 2014, under *ICD-9*, there were 10 133 SA ED visits. Total hospital charges for these visits were more than $233 million in 2019, a 3669.2% increase since 2006 ($6.35 million; AAPC rate, 27.3% [range, 20.5%-34.4%]; *P* < .001).

Most of the SA ED visits were by women (91.5%-92.0% in 2016-2019; 96.1% in 2007), but a growing percentage of visits were by men, increasing from 3.9% of visits in 2007 to 8.0% to 8.5% of visits in 2016-2019 (*P* < .001). Individuals aged 18 to 25 years were overrepresented in the data, accounting for 41.2% (22 784 of 55 296) to 48.6% (2043 of 4200) (*P* < .001) of SA ED visits annually vs 17.6% (15 557 970 of 88 288 926) to 22.2% (16 508 293 of 74 225 760) of all-cause ED visits (Table 1). Individuals in the lowest income quartiles also were consistently overrepresented (Table 2). This disparity peaked in 2010 when 41.3% (2980 of 7225 visits) and 24.8% (1788 of 7225) of SA ED visits were by individuals in the lowest and second-lowest income quartiles (Table 2). In 2019, the lowest income quartile represented 32.8% of SA ED visits (18 108 of 55 296) and 35.9% of all-cause ED visits, whereas the second-lowest income quartile represented 24.9% (13 739 of 55 296) of SA ED visits and 25.5% of all-cause ED visits. Race and ethnicity data were first available in NEDS in 2019. For SA ED visits by race and ethnicity, 760 patients (1.4%) were Asian or Pacific Islander, 11 297 (20.9%) were Black, 6580 (12.2%) were Hispanic, 757 (1.4%) were Native American, and 32 352 (59.8%) were White, compared with 1 786 660 (2.1%) Asian or Pacific Islander, 20 934 397 (24.1%) Black, 13 712 585 (15.8%) Hispanic, 528 342 (0.6%) Native American, and 46 834 060 (53.9%) White patients for all other diagnoses.

**Table 1.** Emergency Department Visits and Admissions Among Adults Aged 18 to 65 Years With Any Diagnosis of Sexual Assault by Age Group

Age group, y	Patients by year, No. (%)													
	2006	2007	2008	2009	2010	2011	2012	2013	2014	2015	2016	2017	2018	2019
**Emergency department visits with any diagnosis of sexual assault**														
18-25	1702 (47.2)	2043 (48.6)	2550 (47.6)	2442 (45.1)	3340 (46.2)	2549 (46.1)	3063 (45.8)	3533 (44.8)	4496 (44.4)	7905 (44.6)	21 417 (44.9)	23 848 (43.6)	22 995 (42.2)	22 784 (41.2)
26-35	979 (27.1)	1162 (27.7)	1340 (25.0)	1505 (27.8)	2106 (29.2)	1586 (28.7)	1947 (29.1)	2215 (28.1)	2911 (28.7)	5388 (30.4)	14 310 (30.0)	16 770 (30.6)	16 374 (30.0)	16 778 (30.3)
36-45	567 (15.7)	625 (14.9)	922 (17.2)	871 (16.1)	1168 (16.2)	772 (14.0)	928 (13.9)	1224 (15.5)	1465 (14.5)	2366 (13.4)	6982 (14.6)	8186 (14.9)	8564 (15.7)	9018 (16.3)
46-65	359 (10.0)	370 (8.8)	546 (10.2)	601 (11.1)	610 (8.4)	619 (11.2)	744 (11.1)	906 (11.5)	1261 (12.4)	2050 (11.6)	5023 (10.5)	5954 (10.9)	6583 (12.1)	6716 (12.1)
Total No. of visits	3607	4200	5357	5420	7225	5526	6681	7878	10 133	17 709	47 732	54 758	54 516	55 296
**Admissions with any diagnosis of sexual assault**														
18-25	87 (19.1)	158 (37.2)	182 (29.8)	132 (27.2)	169 (27.6)	97 (25.3)	140 (33.0)	118 (23.8)	210 (35.3)	96 (19.2)	387 (28.5)	451 (23.8)	422 (22.7)	571 (23.9)
26-35	147 (32.4)	105 (24.7)	125 (20.4)	119 (24.6)	158 (25.8)	72 (18.7)	124 (29.2)	126 (25.5)	156 (26.3)	188 (37.5)	465 (34.2)	521 (27.5)	533 (28.6)	749 (31.3)
36-45	117 (25.8)	83 (19.6)	185 (30.3)	116 (23.8)	149 (24.3)	100 (26.1)	58 (13.7)	133 (26.8)	108 (18.2)	66 (13.2)	217 (16)	523 (27.6)	412 (22.1)	502 (21.0)
46-65	103 (22.7)	78 (18.4)	119 (19.5)	118 (24.4)	136 (22.2)	114 (29.9)	102 (24.1)	118 (23.9)	120 (20.2)	150 (30.0)	290 (21.3)	399 (21.0)	495 (26.6)	567 (23.7)
Total No. of admissions	455	424	612	485	611	383	424	495	595	500	1358	1893	1862	2390

**Table 2.** Emergency Department Visits and Admissions Among Adults Aged 18 to 65 Years With Any Diagnosis of Sexual Assault by Zip Code Income Quartile

Income quartile	Patients by year, No. (%)													
	2006	2007	2008	2009	2010	2011	2012	2013	2014	2015	2016	2017	2018	2019
**Emergency department visits with any diagnosis of sexual assault**														
Lowest	1149 (31.9)	1498 (35.7)	1855 (34.6)	2135 (39.4)	2980 (41.3)	1962 (35.5)	2608 (39.0)	2992 (38.0)	4168 (41.1)	6454 (36.4)	16 668 (34.9)	18 579 (33.9)	19 157 (35.1)	18 108 (32.7)
Second lowest	1085 (30.1)	1020 (24.3)	1608 (30.0)	1414 (26.1)	1788 (24.8)	1077 (19.5)	1803 (27.0)	1806 (22.9)	2559 (25.3)	3940 (22.2)	11 382 (23.8)	13 008 (23.8)	13 933 (25.6)	13 739 (24.8)
Third lowest	703 (19.5)	785 (18.7)	1003 (18.7)	900 (16.6)	1238 (17.1)	1224 (22.1)	1301 (19.5)	1622 (20.6)	1608 (15.9)	3711 (21.0)	9542 (20.0)	11 591 (21.2)	10 295 (18.9)	12 440 (22.5)
Highest	557 (15.4)	770 (18.3)	710 (13.3)	809 (14.9)	939 (13.0)	1073 (19.4)	792 (11.8)	1173 (14.9)	1545 (15.2)	3009 (17.0)	8600 (18.0)	9987 (18.2)	8533 (15.7)	8985 (16.2)
Total No. of visits	3607	4200	5357	5420	7225	5526	6681	7878	10 133	17 709	47 732	54 758	54 516	55 296
**Admissions with any diagnosis of sexual assault**														
Lowest	131 (28.9)	150 (35.4)	223 (36.4)	154 (31.7)	231 (37.7)	147 (38.5)	144 (34.1)	202 (40.7)	216 (36.4)	204 (40.8)	437 (32.2)	637 (33.7)	637 (34.2)	571 (23.9)
Second lowest	114 (25.1)	123 (29.1)	159 (26.0)	137 (28.2)	146 (23.9)	70 (18.3)	117 (27.7)	110 (22.3)	126 (21.2)	84 (16.7)	417 (30.7)	422 (22.3)	524 (28.1)	749 (31.3)
Third lowest	104 (22.9)	75 (17.7)	121 (19.7)	105 (21.7)	118 (19.3)	62 (16.3)	54 (12.8)	84 (17.0)	117 (19.7)	96 (19.2)	248 (18.3)	421 (22.2)	264 (14.2)	502 (21.0)
Highest	84 (18.4)	48 (11.3)	58 (9.4)	57 (11.8)	93 (15.2)	80 (20.9)	77 (18.2)	56 (11.3)	90 (15.2)	88 (17.6)	190 (14.0)	306 (16.2)	269 (14.5)	567 (23.7)
Total No. of admissions	455	424	612	485	611	383	424	495	595	500	1358	1893	1862	2390

Despite the uptick in SA ED visits from 2006 to 2019, admission rates decreased by 8.3%, from 12.6% in 2006 to 4.3% in 2019 (*P* < .001) (Figure 1). The admission rate was lowest at 2.8% in 2015 (Figure 1) in contrast to the 11.0% admission rate for age-matched individuals with all other diagnoses. Older and Medicaid-insured individuals were overrepresented in admissions relative to total SA ED visits. Medicaid-insured individuals, including pregnant women and patients with low income, comprised 27.4% to 53.9% of the patients admitted, despite accounting for only 19.5% to 29.5% of SA ED visits (*P* < .001) (Table 3). Patients aged 46 to 65 years were disproportionately represented in admissions for SA, doubling their proportion of admissions (18.4%-30.0%) relative to visits (8.8%-12.4%) from 2006 through 2019 (*P* < .001) (Table 1). Asian or Pacific Islander, Native American, and White patients were also overrepresented in SA admissions (49 [2.1%], 70 [3.1%], and 1459 [63.7%], respectively; *P* < .01) relative to total SA ED visits (760 [1.4%], 757 [1.4%], and 32 352 [59.8%], respectively).

**Table 3.** Emergency Department Visits and Admissions Among Adults Aged 18 to 65 Years With Any Diagnosis of Sexual Assault by Payer

Payer	Patients by year, No. (%)													
	2006	2007	2008	2009	2010	2011	2012	2013	2014	2015	2016	2017	2018	2019
**Emergency department visits with any diagnosis of sexual assault**														
Medicare	223 (6.2)	198 (4.7)	271 (5.1)	273 (5.0)	452 (6.3)	309 (5.6)	456 (6.8)	474 (6.0)	752 (7.4)	1141 (6.4)	2614 (5.5)	2840 (5.2)	2902 (5.3)	3192 (5.8)
Medicaid	926 (25.7)	832 (19.8)	1115 (20.8)	1060 (19.5)	1827 (25.3)	1492 (27.0)	1851 (27.7)	2091 (26.5)	3019 (29.8)	4648 (26.2)	13 732 (28.8)	13 647 (24.9)	16 108 (29.5)	15 306 (27.7)
Private	1011 (28.0)	1470 (35.0)	1755 (32.8)	1717 (31.7)	2033 (28.1)	1663 (30.1)	1484 (22.2)	2239 (28.4)	2278 (22.5)	5250 (29.6)	12 407 (26.0)	13 251 (24.2)	12 834 (23.5)	13 338 (24.1)
Self-pay	998 (27.7)	1179 (28.1)	1261 (23.5)	1315 (24.3)	1816 (25.1)	1298 (23.5)	1313 (19.6)	1720 (21.8)	1912 (18.9)	2720 (15.4)	8080 (16.9)	9532 (17.4)	10 643 (19.5)	9846 (17.8)
No charge	50 (1.4)	27 (0.6)	49 (0.9)	33 (0.6)	86 (1.2)	64 (1.2)	25 (0.4)	65 (0.8)	58 (0.6)	615 (3.5)	351 (0.7)	1903 (3.5)	538 (1.0)	597 (1.1)
Other	384 (10.6)	448 (10.7)	807 (15.1)	1008 (18.6)	988 (13.7)	669 (12.1)	1539 (23.0)	1264 (16.0)	2068 (20.4)	3090 (17.4)	10 293 (21.6)	13 330 (24.3)	11 170 (20.5)	12 643 (22.9)
Total No. of visits	3607	4200	5357	5420	7225	5526	6681	7878	10 133	17 709	47 732	54 758	54 516	55 296
**Admissions with any diagnosis of sexual assault**														
Medicare	62 (13.6)	54 (12.8)	73 (12.0)	79 (16.2)	94 (15.5)	68 (17.8)	68 (16.0)	86 (17.5)	85 (14.2)	95 (19.0)	165 (12.2)	221 (11.7)	228 (12.2)	304 (12.7)
Medicaid	140 (30.7)	146 (34.4)	246 (40.1)	133 (27.4)	282 (46.2)	114 (29.9)	182 (43)	200 (40.4)	264 (44.4)	190 (37.9)	732 (53.9)	786 (41.5)	940 (50.5)	1123 (47.0)
Private	110 (24.3)	122 (28.7)	99 (16.2)	94 (19.3)	100 (16.4)	103 (27.0)	83 (19.5)	59 (11.8)	126 (21.1)	87 (17.4)	211 (15.6)	342 (18.1)	283 (15.2)	383 (16.0)
Self-pay	92 (20.3)	50 (11.9)	132 (21.6)	110 (22.6)	70 (11.5)	59 (15.3)	50 (11.9)	99 (19.9)	84 (14.1)	74 (14.8)	161 (11.8)	349 (18.4)	215 (11.6)	361 (15.1)
No charge	23 (5.0)	12 (2.8)	17 (2.8)	9 (1.9)	18 (2.9)	11 (2.8)	9 (2.1)	6 (1.1)	3 (0.5)	9 (1.8)	12 (0.9)	33 (1.8)	17 (0.9)	27 (1.1)
Other	28 (6.2)	40 (9.5)	36 (5.9)	58 (12.0)	43 (7.0)	25 (6.4)	27 (6.4)	46 (9.3)	29 (4.8)	35 (6.9)	77 (5.7)	152 (8.1)	154 (8.3)	182 (7.6)
Total No. of admissions	455	424	612	485	611	383	424	495	595	500	1358	1893	1862	2390

**Figure 2.**
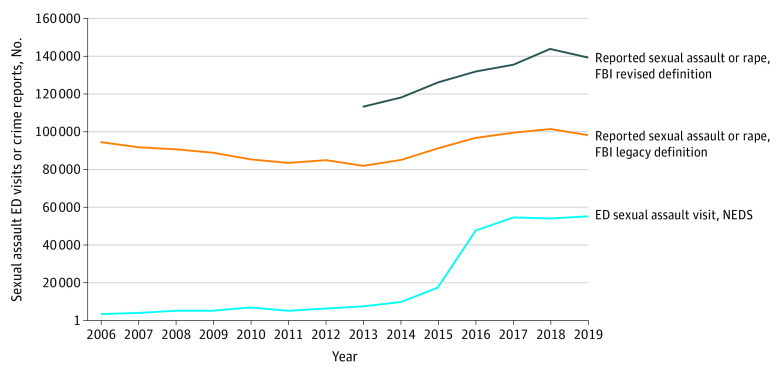
Emergency Department (ED) Visits for Sexual Assault vs Federal Bureau of Investigation (FBI) Uniform Crime Reporting Program–Reported Sexual Assaults, 2006-2019 NEDS, National Emergency Department Sample.

Comparison of the NEDS and FBI databases revealed that ED visits for SA increased more than 1533%, whereas the FBI-reported legacy and revised definitions for SA increased by 7% and 23%, respectively (Figure 2). Data from 2019 revealed that there were still fewer survivors (55 296) seeking ED care compared with reporting to LEAs (98 213 and 139 815 for legacy and revised definitions, respectively).

## Discussion

To our knowledge, this cross-sectional study is the largest longitudinal study of SA ED visits in the US to date. Our analysis of the NEDS database from 2006 through 2019 suggests that adult SA survivors are increasingly presenting to US EDs. The *ICD-9* to *ICD-10* code switch in late 2015 increased coding specificity across all ED diagnoses, including SA. Our subgroup analysis found that the number of confirmed rape/SA cases coded under *ICD-10* in 2015 more than doubled the number reported under *ICD-9* in 2014. There were 25 149 suspected SA ED cases in 2016, possibly because of increased provider comfort with coding for suspected SA. Since 2016, we observed an even distribution of suspected rape/SA visits (50.7%) compared with confirmed rape/SA visits (49.3%). Although the association between increased coding specificity and documentation of SA is still unclear, *ICD-10* likely contributed to increased ED documentation of SA. Other studies performed after the *ICD* switch revealed mixed results. One analysis found that *ICD-10* coding only resulted in fewer diagnoses per patient.^45^ Other reports showed increases in opioid-related stays,^46^ severe maternal morbidity,^47^ and mental health–related presentations under *ICD-10*.^48^ The findings of these studies suggest that the nuanced approach of *ICD-10* may favor increased specificity of coding; however, this is still largely speculative.^45,48,49^ A future validation study could explore how *ICD-9* and *ICD-10* codes are used clinically to address this point.

The data from 2006 through 2015 and from 2016 through 2019 still show steady increases in SA ED visits independent of the *ICD* coding change. Additionally, the increase in FBI-reported rapes/SA from 2015 through 2019 suggests the presence of non-*ICD* factors, including the #MeToo movement and the Larry Nassar/USA Gymnastics case, that may have influenced SA ED visits and LEA reporting (Figure 2). Population growth of adults aged 18 to 64 years by 7% from 2006 to 2019 may have played a role.^50^ Comparison of the NEDS and FBI data revealed increased growth in the rate of SA survivors presenting to EDs relative to LEA reporting. Although the annual FBI rape/SA offense counts were still higher in 2019 than the total SA ED visits, there was a decline in FBI counts compared with 2018, whereas SA ED visits continued to increase (Figure 2). These FBI data provide context for LEA reporting on rape/SA and have previously been used to understand national trends in SA,^51^ gun violence,^52,53^ and relationship aggression.^54^ Future studies could explore other crime databases to validate these trends.

Sexual assault ED visits increased 1533% during 2006-2019, whereas all-cause ED visits increased by 23% during the same period (Figure 1). Although SA still comprises a small proportion of total ED visits at 0.06%, this magnitude of increase suggests that certain factors may be encouraging survivors to seek ED care. This comparison corroborates a previous analysis suggesting no significant change in overall ED diagnoses after the *ICD* code change.^48^ Comparison of hospital admission rates for SA and all-cause ED visits demonstrates a disproportionate decrease for SA-related admissions, suggesting a shift in the population presenting to the ED for SA or factors decreasing the need for admission (Figure 1).

Although young adult women comprised most SA ED visits, male SA survivors represented a growing proportion of these visits. Our findings also uncover disparities with respect to income representation for SA ED visits. Patients from the lowest income quartile by zip code were overrepresented in both SA ED visits and all-cause ED visits, suggesting that these income differences in SA ED use may be driven by the reputation of the ED as a setting for those without equitable health care access. Increased SA ED visits also suggest a gap in access to care elsewhere and an opportunity to enhance ED-based services to meet this population’s needs. Furthermore, the racial distribution of SA ED visits aligned with all-cause ED visits.

Admission data for SA ED visits revealed that patients with lower incomes and government insurance plans are disproportionately admitted. Older survivors (aged 46-65 years) are more likely to be admitted than younger survivors (aged 18-25 years), which may be attributable to comorbid conditions that confer increased risk.^28^ Patients with Medicaid insurance also are overrepresented in admissions relative to their share of SA ED visits. Given that there are no known reimbursement differences that would explain this discrepancy, this finding suggests confounding factors and merits further investigation.

Our findings that SA survivors presenting to EDs were disproportionately younger women align with the existing literature.^13,21,23,24^ Our study also reveals that lower-income patients are disproportionately represented among SA ED visits. Analysis of hospital admission data revealed notable disparities not previously captured, namely that elderly and Medicaid-insured survivors are more likely to be admitted. Comparison with the UCRP database reveals trends associated with medical help seeking after SA vs formal reporting to LEAs. Previous studies have shown that a minority of survivors seek medical care after SA.^9,55^ Law enforcement agency reporting has also been associated with the likelihood that an SA survivor will seek medical help.^9^ These national trends prompt further exploration of factors influencing the increased growth of SA ED use vs LEA reporting.

### Strengths and Limitations

Strengths of this study include its size, national scope, and inclusion of hospital admission data. The breadth of the NEDS database decreases the risk for sampling bias, whereas the national scope of both the NEDS and UCRP databases increases the study’s external validity. To our knowledge, no other studies have offered national comparisons with crime data to contextualize how SA ED use is associated with LEA reporting. Our study also included novel findings with regard to how payer, socioeconomic, and racial variables influence ED use for SA and highlights important disparities that warrant further exploration.

This study has several limitations. Because NEDS is visit based, patients with several ED visits were potentially represented multiple times. The NEDS is also derived from billing codes, so isolated coding errors may be misrepresented. The FBI UCRP data also have limitations, including voluntary reporting from LEAs, narrow definitions for SA, documentation of offense-based data, and an inability to capture unreported SA. The voluntary nature of data contribution to both databases increases potential selection bias. Possible measurement bias also exists due to the imperfect SA definitions that limit the study’s accuracy.

## Conclusions

The findings from this cross-sectional study suggest that adult SA survivors comprise a population increasingly presenting to US EDs. We highlight noteworthy, evolving trends on the demographic characteristics of survivors seeking emergency medical care and how SA ED visits compare with all-cause ED visits. The observed increase in SA ED visits may have been catalyzed by a combination of the switch to *ICD-10* coding, population growth, and contemporary social justice movements such as #MeToo. Strikingly, hospital admissions for SA ED visits have been declining overall. Although the reason for this decline is unclear, possible causes could include differences in evaluation patterns, differences in severity of assault, and/or differences in health system and patient factors.

As few as 21% of survivors seek medical care after SA,^55^ meaning that the survivors captured in this study represent a fraction of total SA-related care needs. Our finding that most SA ED visits are by young, female, and low-income survivors can inform policy changes to better support these individuals. In addition, given that 95% of survivors are ultimately discharged from the ED without admission, we should consider developing outpatient or longitudinal care settings that might better serve all survivors. Medical professionals in EDs and inpatient and outpatient care settings must consider the utilization patterns and survivor populations presented here to better understand emergency medical care seeking for SA. Although medical help seeking is just one piece of the broader picture of SA in the US, we as health care professionals are responsible for supporting survivors in seeking care to prevent unnecessary long-term physical or emotional sequelae. Equitable access for patients and appropriate educational resources for providers are crucial for the path forward. Survivors must be supported and motivated to seek the help they need, desire, and deserve.
